# Supplementary material for: Older women’s experience with COVID-19 pandemic: A study of risk perception and coping among culturally and linguistically diverse population in South Australia
Source: PLoS One. 2024 Mar 28;19(3):e0301366. doi: 10.1371/journal.pone.0301366 (PMC10977767; doi:10.1371/journal.pone.0301366)
Supplement: S1 File — (DOCX) [file pone.0301366.s002.docx]

**Appendix 1: Supplementary File 1.** Survey questionnaire

**Project title:** Self-reported Risk Assessment and Coping Strategies of CALD Older South Australians in COVID-19 Pandemic

**Part A: Demographic questions**

| Gender | Female | Male | Others |
| --- | --- | --- | --- |
| Age (Years) | 60-64 | 65-69 | 70-74 |
|  | 75-79 | 80-84 | 85 ≥ |
| Level of education | No formal education | Primary school | High school |
|  | Bachelors | Masters | Others |
| Ethnicity | ………………………………………….. | | |

**Part B: Questionnaire on the self-risk assessment and risk management strategies**

The following are a series of statements concerning the people’s risk of being affected by, coping with COVID-9. Please indicate to what extent you agree or disagree with these statements. There are 5 possible responses to the following questions:

**Table 1: Risk perception of being infected by COVID-19**

| **Questions** | **Very unlikely (1)** | **Unlikely (2)** | **Neutral (3)** | **Likely (4)** | **Very likely (5)** |
| --- | --- | --- | --- | --- | --- |
| I might become infected by COVID-19 in the near future? | 1 | 2 | 3 | 4 | 5 |
| My family members might become infected by COVID-19 in the near future? | 1 | 2 | 3 | 4 | 5 |
| My friends might become infected by COVID-19 in the near future? | 1 | 2 | 3 | 4 | 5 |

Note: 5-point Likert Scale (1=very unlikely, 5= very likely)

**Table 2: Risk perception of being affected by COVID-19**

| **Dread risk** | **Strongly disagree (1)** | **Disagree (2)** | **Neutral (3)** | **Agree (4)** | **Strongly agree (5)** |
| --- | --- | --- | --- | --- | --- |
| COVID-19 is a global disaster | 1 | 2 | 3 | 4 | 5 |
| COVID-19 will become more dangerous over time | 1 | 2 | 3 | 4 | 5 |
| COVID-19 will affect future generations | 1 | 2 | 3 | 4 | 5 |
| I can easily reduce the risk of infection | 1 | 2 | 3 | 4 | 5 |
| The consequences of COVID-19 for me are my responsibility | 1 | 2 | 3 | 4 | 5 |
| COVID-19 affects me personally | 1 | 2 | 3 | 4 | 5 |
| **Unknown risk** | | | | | |
| COVID-19 is something completely new to me | 1 | 2 | 3 | 4 | 5 |
| The effects of COVID-19 can be managed well | 1 | 2 | 3 | 4 | 5 |
| The experts know about COVID-19 | 1 | 2 | 3 | 4 | 5 |
| I know that I will not be affected by COVID-19 | 1 | 2 | 3 | 4 | 5 |
| **Fear** | | | | | |
| The COVID-19 worries me | 1 | 2 | 3 | 4 | 5 |
| I am afraid of being affected by COVID-19 | 1 | 2 | 3 | 4 | 5 |

Note: 5-point Likert Scale (1=strongly disagree, 5=strongly agree)

**Table 3: Behavioural precautions and emergency preparedness**

| **Behavioural precautions** | | | | | |
| --- | --- | --- | --- | --- | --- |
| I wash and disinfect my hands more often than usual | 1 | 2 | 3 | 4 | 5 |
| I avoid public places/events | 1 | 2 | 3 | 4 | 5 |
| I avoid public transports (tram, bus, train) | 1 | 2 | 3 | 4 | 5 |
| I avoid contact with risk groups (old people and people with previous/current illnesses) | 1 | 2 | 3 | 4 | 5 |
| I bought larger quantities of hand disinfectant/soap | 1 | 2 | 3 | 4 | 5 |
| I bought larger amounts of staple foods (flour, sugar, pasta, rice, canned food) due to COVID-19 | 1 | 2 | 3 | 4 | 5 |
| I bought large quantities of toilet paper and other hygiene items | 1 | 2 | 3 | 4 | 5 |
| **Emergency preparedness** | | | | | |
| I bought more food than usual due to COVID-19 | 1 | 2 | 3 | 4 | 5 |
| I don’t want to go shopping every day | 1 | 2 | 3 | 4 | 5 |
| I buy large quantities of special offers | 1 | 2 | 3 | 4 | 5 |
| I deliberately store essential goods in order to be prepared for COVID-19 | 1 | 2 | 3 | 4 | 5 |
| I collect all emergency services contact details | 1 | 2 | 3 | 4 | 5 |

Note: 5-point Likert Scale (1=strongly disagree, 5=strongly agree)
