# Supplementary material for: Older women’s experience with COVID-19 pandemic: A study of risk perception and coping among culturally and linguistically diverse population in South Australia
Source: PLoS One. 2024 Mar 28;19(3):e0301366. doi: 10.1371/journal.pone.0301366 (PMC10977767; doi:10.1371/journal.pone.0301366)
Supplement: Attachment — Submitted filename: PONE-D-23-24279_reviewer.docx [file pone.0301366.s003.docx]

The paper examines the issue of experiences of COVID-19 in CALD older women in South Australia, particularly their risk perception and coping strategies. This is an important study. However, there are significant issues the authors would need to address before this paper can be published. The major concerns are around:

1. Handling of missing data. how many participants were dropped? How the missing data for the remaining participants were handled? It would also be important to highlight more clearly about this, given the small sample size in this study.
2. Analysis of outcome variables. How did the author analyse the outcome variables? Did they score the mean or sum the scores for each item? If so, supported with evidence from the literature, are there baselines or thresholds that indicate negative or positive experiences?
3. Did the authors assess the data to ensure it met the assumptions for the independent sample t-test? Further information is required.
4. Qualitative analysis. Who analysed the qualitative data? How many people were involved in the analysis? How were divergences resolved if two or more people were involved in the analysis? How long did the interview last? Where were the interviews done? Were these recorded? transcribed? If so, how were they done? Did the author use any software program to help with this qualitative analysis?

General considerations around the following points also need to be considered. The paper requires careful revision for:

1. grammar
2. use of language. For example, “native Australians” is rarely used to describe Australia’s First Nations. Consider using Australia’s First Nations, Aboriginal and Torres Strait Islanders, or Indigenous Australians.
