# Supplementary material for: Older women’s experience with COVID-19 pandemic: A study of risk perception and coping among culturally and linguistically diverse population in South Australia
Source: PLoS One. 2024 Mar 28;19(3):e0301366. doi: 10.1371/journal.pone.0301366 (PMC10977767; doi:10.1371/journal.pone.0301366)
Supplement: Attachment — Submitted filename: Plos Oct 2023.docx [file pone.0301366.s005.docx]

To

The Author,

Thanks for the submission and choosing older CALD women as the subject.

However, I have some observations:

1. The detail of the data collection procedure should be mentioned.
2. What is the time of the survey?
3. Is it reliable to use online survey among older CALD women to collect data?
4. Among 109 participants, only 25 patients agreed for the telephone interview and 10 of them were unavailable or could not be reached. This is the weakest part of this paper.
5. Are there any significant differences of the risk perception and coping among the older CALD women and men? If yes, please mention those. And if not, why do the older men were excluded from this study? Please explain.

In its current format, I will not recommend this paper for publication.
