# Supplementary material for: Older women’s experience with COVID-19 pandemic: A study of risk perception and coping among culturally and linguistically diverse population in South Australia
Source: PLoS One. 2024 Mar 28;19(3):e0301366. doi: 10.1371/journal.pone.0301366 (PMC10977767; doi:10.1371/journal.pone.0301366)
Supplement: Attachment — Submitted filename: Response to reviewers Plos One.docx [file pone.0301366.s006.docx]

17 November 2023

**To**

**Editor**

**PLOS ONE**

RE: Ms. No. PONE-D-23-24279 - Older women experience with COVID-19 pandemic: A study of risk perception and coping among culturally and linguistically diverse population in South

Australia.

Dear Editor,

Thank you for the opportunity to strengthen our manuscript submission. We found the review comments positive and helpful and have addressed them in the point-by-point table below. A point-by-point response letter has been accompanied with our revised manuscript with track changes and clean version of the final manuscript.

This letter provides a detailed response to each reviewer point raised, describing exactly what amendments have been made to the manuscript text and where these can be viewed. All changes to the manuscript are indicated in the text by highlighting or using track changes. We thank you for your time and look forward to hearing from you regarding our manuscript.

| **Reviewer 1** | |
| --- | --- |
| The paper examines the issue of experiences of COVID-19 in CALD older women in South Australia, particularly their risk perception and coping strategies. This is an important study. However, there are significant issues the authors would need to address before this paper can be published. The major concerns are around:  Handling of missing data. how many participants were dropped? How the missing data for the remaining participants were handled? It would also be important to highlight more clearly about this, given the small sample size in this study. | The survey and analysis section has been reviewed as follows:  In the dataset comprising 109 participants, the surveys scales were missing for 6.42% (n=7) participants. To address this, mode imputation was applied to fill the missing cases, with a separate imputation for each indicator. |
| 1. Analysis of outcome variables. How did the author analyse the outcome variables? Did they score the mean or sum the scores for each item? If so, supported with evidence from the literature, are there baselines or thresholds that indicate negative or positive experiences? | This survey and analysis section has been reviewed to avoid confusion.  Mean-sum-scores were used in the analysis:  Mean sum-score of coping was 79.8 (SD: 9.3), indicating agreement with the four items of coping behaviors.  And  Mean sum-score of emergency preparation was 23.9 (SD: 6.8) indicating a neutral position with the 8 indicators of emergency preparation.  Our study focused on comparing the participants’ agreements with coping and emergency preparation across their demographics. |
| 2. Did the authors assess the data to ensure it met the assumptions for the independent sample t-test? Further information is required. | The following information has been added to the statistical analysis section to address this review comment:  Outliers were checked, and assumptions for parametric analysis were verified in the data.  …  Independent-sample t-test was used (as the independent variables had two categories) to compare coping and preparation scores across demographics |
| 3. Qualitative analysis. | As requested, we have added detail as appropriate. |
| a. Who analysed the qualitative data? | This has been added, “HM, EP, conflicts resolved by MH”. |
| b. How many people were involved in the analysis? | Three is obvious based on the previous response. |
| c. How were divergences resolved if two or more people were involved in the analysis? | Resolved by a third person, as in previous comments. |
| d. How long did the interview last? | While much of this is stated in Methods/*Research design*, we have also added to the to the Methods/*In-depth Interviews: data and analysis* subsection, “Individual telephone interviews were approximately one-hour in duration conducted during November 2020-March 2021 with 15 women who had self-nominated to calls for interview participants or upon via the completion of their survey.“ |
| e. Where were the interviews done? |  |
| f. Were these recorded? transcribed? If so, how were they done? | We originally stated, “Interviews were audio recorded and transcribed verbatim.” |
| g. Did the author use any software program to help with this qualitative analysis? | We added, “Each step of the analysis was undertaken manually by two researchers (HM, EP) and conflicts resolved by a third researcher (MH).” |
| General considerations around the following points also need to be considered. The paper requires careful revision for:   1. grammar | A native English-speaking author has corrected grammar throughout. |
| 2. use of language. For example, “native Australians” is rarely used to describe Australia’s First Nations. Consider using Australia’s First Nations, Aboriginal and Torres Strait Islanders, or Indigenous Australians. | This has been corrected to “native born Australians”. We have also added to the CALD comparison description “irrespective whether they are first- or second-generation immigrants” to make this clear that we are not referring to Australia’s first Nations peoples. |
| **Reviewer 2** | |
| 1. I have also made some comments on the paper for you to consider. | Thank you, we have worked thought these comments and responded as requested by this reviewer. |
| Comment from the paper re “The coping with disasters scale has two sections; (i) emotional and behavioural precaution, and (ii) emergency preparedness, constructed based on Lazarus & Folkman’s (1984) coping strategies. The scale  includes 23 emotional and behavioural strategies and eight emergency preparation ways. In addition to 31 separate indicators, two sum-scores (23 items of coping and 8 items of emergency preparation) were considered.”   a. How did the author analyse the outcome variables? Did they score the mean or sum the scores for each of the items? If so, is there a baseline or threshold which indicates negative or positive experience? | This section has been reviewed to avoid confusion.  Mean-sum-scores were used in the analysis:  Mean sum-score of coping was 79.8 (SD: 9.3), indicating agreement with the four items of coping behaviors.  And  Mean sum-score of emergency preparation was 23.9 (SD: 6.8) indicating a neutral position with the 8 indicators of emergency preparation.  Our study focused on comparing the participants’ agreements with coping and emergency preparation across their demographics. |
| Comment from the paper re – “Independent-sample t-test was used to compare coping and preparation scores across demographics. Sampling “   b. How did the authors score the measured items? Did the authors assess the data to ensure it met the assumptions for independent sample t-test? Further information is required | The following information has been added to the statistical analysis section to address this review comment:  Outliers were checked, and assumptions for parametric analysis were verified in the data.  …  Independent-sample t-test was used (as the independent variables had two categories) to compare coping and preparation scores across demographics |
| Comment from the paper re – “the theme were extracted for narrating.”   c. How was this done? did the author use any software program to help with this qualitative analysis? How many people were involved in the thematic analysis? Who were the authors? If more than two people were involved in the analysis, how were divergences in the analysis resolved?” | This has been revised per Reviewer #1 above (it is not clear whether this is the same reviewer responding on the paper as in comments by reviewer #1). |
| Comment from the paper re – “Not surprisingly, 92.7% participants were not involved in the formal labor force.”   d. Not intuitive, especially when participants are older. consider removing. | Sentence removed. |
| Comment from the paper re age group 60-64 in Table 1   e. This include people who are not retired. Consider including retirees only. It may be accurate to describe people in the working age group as 'older' to avoid running the risk of ageism | We have specified in response to an earlier comment re ‘older’ and clarified our use of >60 years based on UN age definition. We are not revising the age groups in our sample considering that there is no retirement age for women in Australia unless a member of judiciary (pension eligibility age for women is 67), and we cannot identify retirees in our sample.  We have also corrected an erratum in which we referred to ‘Elderly’ – changed to consistent use of ‘older’ considering the UN definition as stated. |
| Comment from paper re quotes.   f. Are these quotes taken from one participant? consider attributing quotes to participants. | Pseudonym names and age of the participants have been added in brackets after each quote |
| Comment from paper re qualitative lead-in.   g. How many few is few? In qualitative research, the focus is on the richness and depth of information. consider using other terms. for example instead of saying few say three people or two people etc. | As a team of researchers, we have members skilled in qualitative analysis who disagree. Since the emphasis is on richness and depth, numbers may be used. However it is common for qualitative reporting to use terms such as ‘few’, ‘some’, ‘many’, etc. |
| 2. The methodology section should be more elaborate. | We have elaborated on the methodology, as in our feedback to Reviewer #1. We have also commenced the methods section with providing research ethics approval and ethics convention. |
| 3. Time of the survey should be mentioned precisely. | We have added in Methods/*Research design*, “Survey and interview data was collected concurrently from July to December 2020.” |
| 4. Manuscript is written in standard English. | Yes |
| 5. I am not a statistician, so I am unable to comment on statistical analysis part. | We have checked the statistical analysis and responded to the other reviewers’ comments accordingly. |
| **Reviewer 3** | |
| Thanks for the submission and choosing older CALD women as the subject.  However, I have some observations:   1. The detail of the data collection procedure should be mentioned. | We have elaborated on data collection per the previous reviewer comments. |
| 2. What is the time of the survey? | Time of survey has been added per previous reviewer comments. |
| 3. Is it reliable to use online survey among older CALD women to collect data? | We have added to limitations, “Recent research has concluded that web-surveys are feasible and a suitable alternative for surveying older people (Kelfve et al, 2020), however we acknowledge that by not offering paper-based or different language alternatives that this may bias results by excluding subsamples who may lack digital or English literacy. Data recruitment and collection methods were impacted by COVID-19 restrictions, limiting opportunity for distribution of materials via public meetings and snowballing, which will have implicated survey and interview response rates.” |
| 4. Among 109 participants, only 25 patients agreed for the telephone interview and 10 of them were unavailable or could not be reached. This is the weakest part of this paper. |  |
| 5. Are there any significant differences of the risk perception and coping among the older CALD women and men? If yes, please mention those. And if not, why do the older men were excluded from this study? Please explain. | This study is focused on women’s experiences. It is not a comparative study and men were not ‘excluded’. We already provided a justification for our study of CALD women based on literature cited which indicates they may be at heightened risk of poor coping. Accordingly, we have removed an erratum in the Methods, *Research design*, where we stated ‘subsample’ which is now corrected to “sample of older CALD women”. |

**Kind regards**

Dr Mohammad Hamiduzzaman

Research Fellow

The University of Sydney

NSW, Australia.
