# Supplementary material for: Older women’s experience with COVID-19 pandemic: A study of risk perception and coping among culturally and linguistically diverse population in South Australia
Source: PLoS One. 2024 Mar 28;19(3):e0301366. doi: 10.1371/journal.pone.0301366 (PMC10977767; doi:10.1371/journal.pone.0301366)
Supplement: Attachment — Submitted filename: Revision 2_ Response to review comments.docx [file pone.0301366.s007.docx]

11 March 2024

**To**

**Editor**

**PLOS ONE**

RE: Ms. No. PONE-D-23-24279 - Older women experience with COVID-19 pandemic: A study of risk perception and coping among culturally and linguistically diverse population in South

Australia.

Dear Editor,

Thank you for the opportunity to strengthen our manuscript submission. We found your comments positive and helpful and have addressed them in the point-by-point table below. A point-by-point response letter has been accompanied with our revised manuscript with track changes and clean version of the final manuscript.

| **Editor comments** | |
| --- | --- |
| 1- Could you add an operational definition of Culturally and linguistically diverse (CALD) to the manuscript? Have a look at at how Australian Institute of Health and Welfare defines CALD: https://www.aihw.gov.au/getmedia/f3ba8e92-afb3-46d6-b64c-ebfc9c1f945d/aihw-aus-221-chapter-5-3.pdf.aspx | The operational definition of CALD has been added. |
| 2- I didn't like the term Non-English speaking Europeans. Almost all the respondents were from Non-English speaking background so there is no need to make this distinction. | The manuscript has been reviewed to address your concern of using Non-English Speaking Europeans. |
| 3- The abstract uploaded on the portal states that 67% women were non-English speaking CALD and they were still provided with English language questionnaire? Were interviews also conducted in English? Why? | The language used for both survey and interviews was English. This was made possible by the fact that the participants have had some level of English proficiency, even though their spoken language at home was not English. This is one of the limitations of this study. |
| 4- I guess it would be better to say that the participants had somewhat English proficiency, but their spoken language at home was not English. Please fix this throughout the manuscript. If you add an operational definition of CALD, it would rectify this confusion. | An operational definition of CALD has been added to avoid confusion. |
| 5- Native-Australians term in confusing. Instead of writing CALD vs native-Australians, could you make it CALD vs. non-CALD throughout the manuscript. Native literally means 'born somewhere', so it can be confused with other people born in Australia, who could be anyone, may be First Nation Australians, Anglo-Australians or CALD Australians. | Thank you for your suggestions. Changes have been made throughout the manuscript. |

**Kind regards**

Dr Mohammad Hamiduzzaman

Research Fellow, School of Health Sciences

The University of Sydney

NSW, Australia.
